# Cell-free synthesis of proteins with selectively ^13^C-labelled methyl groups from inexpensive precursors

**DOI:** 10.5194/mr-4-187-2023

**Published:** 2023-07-19

**Authors:** Damian Van Raad, Gottfried Otting, Thomas Huber

**Affiliations:** 1 Research School of Chemistry, Australian National University, Canberra, ACT 2601, Australia; 2 ARC Centre of Excellence for Innovations in Peptide & Protein Science, Research School of Chemistry, Australian National University, Canberra, ACT 2601, Australia

## Abstract

The novel eCell system maintains the activity of the entire repertoire of metabolic *Escherichia coli* enzymes in cell-free protein synthesis. We show that this can be harnessed to produce proteins with selectively ${}^{13}$ C-labelled amino acids from inexpensive ${}^{13}$ C-labelled precursors. The system is demonstrated with selective ${}^{13}$ C labelling of methyl groups in the proteins ubiquitin and peptidyl-prolyl *cis–trans* isomerase B. Starting from 3- ${}^{13}$ C-pyruvate, ${}^{13}$ C-HSQC cross-peaks are obtained devoid of one-bond ${}^{13}$ C– ${}^{13}$ C scalar couplings. Starting from 2- ${}^{13}$ C-methyl-acetolactate, single methyl groups of valine and leucine are labelled. Labelling efficiencies are 70 % or higher, and the method allows us to produce perdeuterated proteins with protonated methyl groups in a residue-selective manner. The system uses the isotope-labelled precursors sparingly and is readily scalable.

## 1 Introduction

The NMR resonance assignments of high molecular-weight proteins critically depend on the availability of samples enriched with stable isotopes (Tugarinov et al., 2006). Conventional strategies based on uniformly ${}^{13}$ C-enriched proteins usually employ [U- ${}^{13}$ C]-glucose as the (de facto) only carbon source in minimal media (Ohki and Kainosho, 2008; Filipp et al., 2009). The ${}^{13}$ C enrichment of proteins enables the sensitive recording of heteronuclear correlation spectra such as 2D ${}^{13}$ C-HSQC spectra, which are particularly sensitive to methyl groups. Methyl groups play a privileged role in the NMR analysis of large protein systems in solution, as their signals can be observed for macromolecular complexes as large as 1 MDa (Boswell and Latham, 2018). Methyl-bearing amino acids are abundant not only in the hydrophobic core of globular proteins but also in hydrophobic ligand binding pockets (Otten et al., 2010). Methyl groups thus serve as useful probes for the analysis of protein structure, dynamics and function (Schütz and Sprangers, 2020).

Among the amino acids with methyl groups, the spectral regions of the methyl groups of isoleucine, leucine and valine (ILV) overlap in a ${}^{13}$ C-HSQC spectrum (Rasia et al., 2012). This poses a problem for large proteins, which not only contain many methyl groups but also feature broad NMR signals (Lange et al., 2012). Furthermore, uniformly ${}^{13}$ C-labelled proteins feature ${}^{13}$ C– ${}^{13}$ C couplings, in particular one-bond ${}^{13}$ C– ${}^{13}$ C couplings ( ${}^{1}J_{\mathrm{CC}}$ ), which lead to broad multiplets in the ${}^{13}$ C dimension of ${}^{13}$ C-HSQC spectra. Several strategies have been devised to resolve the methyl cross-peaks of ILV residues.

i. Protein samples can be produced from amino acid mixtures containing only a single amino acid with isotope enrichment. Suitably labelled amino acids are available commercially but can be expensive (Kainosho and Güntert, 2009; Takeda et al., 2010). In many cases, the most affordable versions of ${}^{13}$ C-labelled amino acids are uniformly enriched with ${}^{13}$ C, which retains the problem of ${}^{13}$ C– ${}^{13}$ C couplings.
ii. As a compromise between cost and selectivity, selectively ${}^{13}$ C-labelled late-stage precursors such as 2-ketobutyrate or 2-ketoisovalerate can be supplied (Goto et al., 1999; Hajduk et al., 2000; Lazarova et al., 2018), which are key intermediates of the biosynthesis of ILV amino acids (Lundström et al., 2007). These precursors are commercially available in selectively ${}^{13}$ C- and ${}^{2}$ H-labelled form to produce proteins with single ${}^{13}$ CH ${}_{3}$ groups in valine, leucine and the $\delta_{1}$ position of isoleucine in an otherwise perdeuterated background and have proven extraordinarily useful for NMR investigations of high molecular-weight proteins (Tugarinov and Kay, 2005). Precursors close to the final stages of amino acid biosynthesis present a cost-efficient way for labelling proteins with high selectivity (Kasinath et al., 2013; Schörghuber et al., 2018) and, by virtue of specific chemical synthesis, solve the problem of ${}^{13}$ C– ${}^{13}$ C couplings.
iii. An elegant extension of methyl labelling is presented by the provision of 2- ${}^{13}$ C-methyl acetolactate in the growth medium, which achieves stereospecific-selective labelling of single methyl groups of valine and leucine (Gans et al., 2010). This approach relies on the activity of several enzymes in the biosynthesis pathways for leucine and valine and thus requires in vivo protein production and, consequently, relatively large quantities of the expensive precursor.
iv. One-bond ${}^{13}$ C– ${}^{13}$ C couplings in uniformly ${}^{13}$ C-labelled proteins can also be removed by NMR techniques. For example, ${}^{13}$ C– ${}^{1}$ H correlation spectra can be recorded with homonuclear ${}^{13}$ C decoupling in the ${}^{13}$ C dimension, either by recording as a constant-time experiment (Vuister and Bax, 1992) or band-selective decoupling (Behera et al., 2020). However, constant-time experiments sacrifice sensitivity and band-selective decoupling of methyl carbons cannot decouple the ${}^{13}$ C multiplet of leucine methyls as the ${}^{13}$ C chemical shifts in their coupling partners are too close.

Selective methyl labelling by the use of late-stage precursors has become one of the most important approaches for NMR studies of large proteins, having been successfully applied to protein complexes up to 1 MDa (Sprangers and Kay, 2007). The cost of late-stage precursors, however, can become significant when the assignment of the methyl cross-peaks can only be obtained by site-directed mutagenesis. A case in point is the 468 kDa multimeric aminopeptidase PhTET2, where the assignment of the alanine C ${}^{\beta }$ H ${}_{3}$ and isoleucine C ${}^{\delta }$ H ${}_{3}$ groups alone consumed 3.2 L of media with expensive ${}^{13}$ C-labelled precursors (Amero et al., 2011). The present work explored the possibility of using earlier precursors of amino acid biosynthesis to produce proteins with ${}^{13}$ CH ${}_{3}$ groups free from one-bond ${}^{13}$ C– ${}^{13}$ C couplings and with the option of a background of perdeuteration.

The optimal labelling scheme should be amenable to cell-free protein synthesis (CFPS), which uses isotope-labelled compounds sparingly (Torizawa et al., 2004). Unfortunately, the biosynthesis of ${}^{13}$ C-labelled amino acids is compromised in in vitro protein expression systems (Linser et al., 2014), although a limited degree of metabolism can be restored by re-introducing certain cofactors (Jewett et al., 2008). For example, metabolites from glycolysis can be used for energy generation in CFPS if cofactors such as NAD ${}^{+}$ and CoA are provided (Kim and Swartz, 2001). Energy generation systems have also been based on phosphoenol pyruvate (PEP) as well as pyruvate, glucose and maltodextrin (Caschera and Noireaux, 2015). In our hands, these systems proved to be more difficult to establish presumably because of their dependence on the activity of multiple enzymes from the glycolytic pathway.

An alternative CFPS approach to proteins with selectively ${}^{13}$ C-labelled ILV residues supplements the reaction with the enzymes required to convert chemically synthesized precursors to the final amino acid. This has been demonstrated with 2-ketoisovalerate and 4-methyl-2-oxovalerate, adding purified aminotransferase IlvE to catalyse the last step in the biosynthesis to valine and leucine, respectively (Lazarova et al., 2018). Conducting the CFPS reaction with an earlier precursor such as methyl acetolactate, however, would require additional enzymes to be active.

The recently established eCell system solves the problem of maintaining the activity of enzymes required for energy regeneration in CFPS (Van Raad and Huber, 2021). Here we show that eCells also conserve the activity of biosynthetic pathways required for amino acid synthesis from simple precursors. eCells are bacterial cells coated with polymers, where the cell wall has been lysed (Van Raad and Huber, 2021). The resulting cells can no longer replicate, but they still contain all bio-macromolecules required for protein synthesis, while their porous polymer coat gives low molecular-weight compounds free access to the cytosol. eCells thus are ideal vehicles for CFPS. We hypothesized that eCells preserve the activity of all enzymes involved in amino acid biosynthesis and therefore allow the production of methyl-labelled amino acids from inexpensive precursors such as 3- ${}^{13}$ C-pyruvate or ${}^{13}$ C-glucose. In the following we demonstrate the excellent utility of eCells to produce proteins with selectively ${}^{13}$ C-labelled methyl groups in valine and leucine made from pyruvate, 2-methyl-4-acetolactate and glucose.

**Figure 1:**
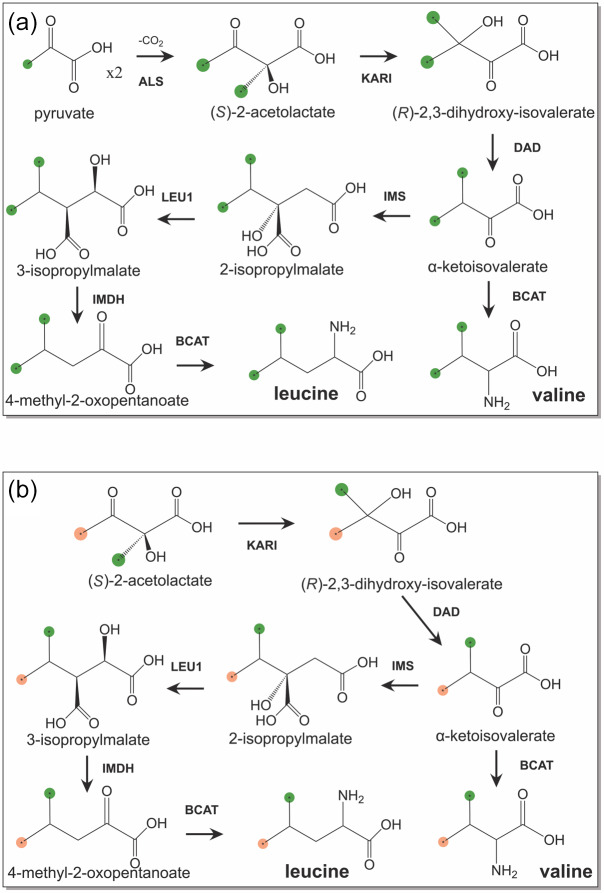
Biosynthetic pathways of leucine and valine from isotope-labelled precursors. ${}^{13}$ C-labelled methyl groups are identified by green balls, and methyl groups at natural isotopic abundance are highlighted by orange balls. **(a)** Biosynthetic pathway starting from 3- ${}^{13}$ C-labelled pyruvate. **(b)** Stereoselective biosynthetic pathway starting from ( S) -2-acetolactate. Abbreviations used: KARI, ketol-acid reductoisomerase; DAD, dihydroxy-acid dehydratase; IMS, 2-isopropylmalate synthase; LEU1, 3-isopropylmalate dehydratase; IMDH, 3-isopropylmalate dehydrogenase; BCAT, branched-chain aminotransferase.

## 2 Materials and methods

### 2.1 Materials

The polyelectrolytes low molecular-weight chitosan (50 000–190 000 Da) and sodium alginate were purchased from Merck. The ethyl ester of 2- ${}^{13}$ C-methyl-4- ${}^{2}$ H ${}_{3}$ -acetolactate (ethyl-2-hydroxy-2- ${}^{13}$ C-methyl-3-oxobutanoate) was purchased from Cambridge Isotope Laboratories (CIL; USA). Perdeuterated amino acids were from CIL and Martek Isotopes (USA); 3- ${}^{13}$ C-pyruvate was from Sigma-Aldrich.

### 2.2 Plasmids

A plasmid was constructed with the pCloDF13 origin of replication, the gene of the *Escherichia coli* peptidyl–prolyl *cis–trans* isomerase PpiB with C-terminal His ${}_{6}$ -tag under control of the T7 promoter and a spectinomycin resistance gene, generating the plasmid pCDF PpiB CTH. For ubiquitin expression a plasmid was constructed with a pCloDF13 origin of replication, the spectinomycin resistance gene and the gene of ubiquitin under control of the T7 promoter (plasmid pCDF Ubi CTH). A *lac* operator was inserted in front of the T7 promoter to reduce background protein expression prior to induction, which reduces the ${}^{13}$ C-labelling efficiency.

### 2.3 Production of eCells

*E. coli* XjB(DE3) ${}^{*}$ cells were transformed with either pCDF Ubi CTH or pCDF PpiB CTH and grown in an LB medium at 37 ${}^{\circ }$ C in baffled flasks with shaking at 180 rpm. Endolysin production was induced at the time of inoculation with a final concentration of 3 mM arabinose. Cells were grown to OD ${}_{600}=0.6$ , harvested by centrifugation at 2773 g and washed three times with 20 mL PBS-E buffer (phosphate-buffered saline with 1 mM EDTA, pH 7.4). For coating with chitosan, 1 g of the cells was resuspended in 20 mL of 0.25 mg mL ${}^{-1}$ chitosan in PBS-E with vigorous shaking for 20 min. A total of 1 g of the cell pellet was washed with 20 mL PBS-E pH 6.0 three times to remove excess chitosan and then resuspended in 20 mL of 0.25 mg mL ${}^{-1}$ alginate PBS-E solution and subjected to vigorous shaking for 20 min. The cells were then washed three times with 20 mL PBS-E pH 6.0, resuspended in PBS-E pH 7.4 and stored at - 80 ${}^{\circ }$ C. eCell weights reported for different CFPS reactions refer to the sedimented pellet of encapsulated cells following decanting of the wash buffer.

### 2.4 Production of deuterated eCells

A total of 5 g sodium pyruvate was dissolved in 50 mL D ${}_{2}$ O and the pH adjusted with 0.1 mM KOD to pH 11. The solution was stirred overnight at 95 ${}^{\circ }$ C to exchange the protons of pyruvate for deuterium. A total of 500 mL M9 minimal media was prepared in D ${}_{2}$ O with 22 mM KH ${}_{2}$ PO ${}_{4}$ , 42 mM Na ${}_{2}$ HPO ${}_{4}$ , 8.6 mM NaCl, 18.6 mM NH ${}_{4}$ Cl, 500 µ L 1 mg mL ${}^{-1}$ thiamine (vitamin B6), 0.1 mM CaCl ${}_{2}$ , 250 µ L 1000 × metal mixture (50 mM FeCl ${}_{3}$ , 10 mM MnCl ${}_{2}$ , 10 mM ZnSO ${}_{4}$ , 2 mM CoCl ${}_{2}$ , 2 mM CuCl ${}_{2}$ and 2 mM NiCl ${}_{2})$ , 5 mM MgSO ${}_{4}$ , 3 mM arabinose and 25 mg mL ${}^{-1}$ spectinomycin. The H–D exchange in pyruvate was confirmed by NMR. The deuterated pyruvate was added to the dry mixture of buffer salts and the final pyruvate-M9 medium made up to 500 mL, adjusted to pH 7.2 and filter-sterilized prior to inoculation.

XjB(DE3) ${}^{*}$ cells that had been transformed with pCDF PpiB CTH were trained for the production of perdeuterated proteins in a protocol adapted from that reported by Li and Byrd (2022). A total of 15 mL of an overnight starter culture of pCDF PpiB CTH was diluted with 15 mL of deuterated pyruvate-M9 medium and incubated at 37 ${}^{\circ }$ C with shaking at 180 rpm. When the OD ${}_{600}$ reached 1.0, the cells were again diluted with 30 mL of deuterated pyruvate-M9 medium and incubated a second time. Upon reaching OD ${}_{600}=1.0$ , the 60 mL culture was spun down, the cells transferred to a 50 mL culture and growth continued overnight at 37 ${}^{\circ }$ C with shaking at 180 rpm. The 50 mL culture was added to 400 mL of deuterated pyruvate-M9 medium and left to grow until OD ${}_{600}=0.75$ was reached, after which the cells were encapsulated as described in Sect. 2.3.

### 2.5 CFPS systems

The protocol for pyruvate-based CFPS was adapted from the phosphate recycling system by Jewett and Swartz (2004). The CFPS buffer contained 0.9 mM UTP and CTP, 50 mM HEPES, 1.5 mM GTP, 1.5 mM ATP, 0.68 µ M folinic acid, 0.64 mM cAMP, 1.7 mM DTT, 3.5 mM of each amino acid (apart from the amino acid(s) to be synthesized by the eCells for isotope enrichment), 60 mM K-Glu, 8 mM Mg-Glu, 2 % v/v PEG-8000, 4 mM sodium oxalate, 0.25 mM CoA, and 0.33 mM NAD ${}^{+}$ . A Roche cOmplete™ Mini protease inhibitor cocktail was added to the CFPS buffer. Of the volume following dissolution of one tablet in 10 mL water, 10 % was added to the CFPS reaction. The reaction was conducted with 33 mM pyruvate.

The protocol for glucose-based CFPS was likewise adapted from the previously published phosphate recycling system (Jewett and Swartz, 2004). The glucose CFPS buffer contained the same components as the pyruvate-based CFPS protocol but with 10 mM sodium phosphate dibasic pH 7.5 and without sodium oxalate and pyruvate. The reaction was conducted with 30 mM glucose.

The CFPS system using creatine phosphate and creatine kinase as an energy source contained the same components as the pyruvate-based CFPS protocol but without sodium oxalate, pyruvate, CoA or NAD ${}^{+}$ and adding 250 µ g mL ${}^{-1}$ creatine kinase, 80 mM creatine phosphate and 6 mM Mg-Glu instead of 8 mM Mg-Glu.

Following the addition of the isotopically enriched precursor, the CFPS buffers for each of these reactions were adjusted to pH 7.5. Frozen aliquots of encapsulated cells were thawed and resuspended in CFPS buffer. The buffer volumes ranged between 5 and 25 mL depending on the weight of cell pellet used (300 mg–1 g). Lysis of the cell wall occurs spontaneously during thawing (Van Raad and Huber, 2021). CFPS for each experiment was conducted at 37 ${}^{\circ }$ C overnight with shaking at 180 rpm.

### 2.6 Acetolactate labelling

By incubating in H ${}_{2}$ O with 0.1 M NaOH (NaOD for deuteration experiment) (pH 13) at 37 ${}^{\circ }$ C for 30 min, 2- ${}^{13}$ C-methyl-4- ${}^{2}$ H ${}_{3}$ -acetolactate as the source for prochiral methyl groups was set free from the ethyl ester. The compound was tested both in pyruvate-based CFPS and in CFPS with the creatine phosphate and creatine kinase system. The CFPS reaction was conducted in 15 mL buffer with 0.1 mM NADP ${}^{+}$ , 3.5 mM 2- ${}^{13}$ C-methyl-4- ${}^{2}$ H ${}_{3}$ -acetolactate and 0.2 mM penoxsulam to inhibit the acetolactate synthase (ALS) enzyme. Ubiquitin was produced from 300 mg eCells and purified using His-Gravitrap columns (GE Healthcare, USA).

For perdeuterated CFPS, all buffer stocks were dissolved in D ${}_{2}$ O, and the pH was adjusted with KOD to pH 7.2. The creatine-phosphate-based CFPS reaction was conducted in 20 mL D ${}_{2}$ O buffer with 5 mM 2- ${}^{13}$ C-methyl-4- ${}^{2}$ H ${}_{3}$ -acetolactate, 0.1 mM NADP ${}^{+}$ , 1 mM of all amino acids in perdeuterated form excluding valine and 0.2 mM penoxsulam to inhibit the acetolactate synthase (ALS) enzyme. PpiB was produced from 800 mg eCells and purified using His-Gravitrap columns (GE Healthcare, USA).

### 2.7 Labelling with 3- ${}^{13}$ C-pyruvate or 1- ${}^{13}$ C-glucose

Dry 3- ${}^{13}$ C-pyruvate was added to 15 mL CFPS buffer at 33 mM final concentration. Leucine, valine and isoleucine were omitted from the amino acid mixture to allow for ${}^{13}$ C labelling of their methyl groups. Ubiquitin and PpiB were expressed using 300 mg eCells and purified using His-Gravitrap columns. To illustrate the scalability of the reaction, ubiquitin samples were also produced with specific labelling of alanine and valine in 5 mL CFPS buffer using 300 mg eCells with the amino acid of interest omitted from the amino acid mixture. PpiB with ${}^{13}$ C-labelled valine was produced in 20 mL pyruvate-based CFPS buffer with 1 g eCells with valine omitted from the amino acid mixture.

To test the performance of 1- ${}^{13}$ C-glucose as ${}^{13}$ C source, dry 1- ${}^{13}$ C-glucose was added to 5 mL glucose-based CFPS buffer at 30 mM final concentration. Leucine and valine were omitted from the amino acid mixture to allow for labelling of their methyl groups. To assess the potential of glutamate in the buffer in diluting the ${}^{13}$ C-label, reactions were conducted with a buffer containing 60 mM K-Glu and 8 mM Mg-Glu or 100 mM adipic acid and 8 mM MgCl ${}_{2}$ .

### 2.8 NMR spectroscopy and isotope labelling yields

All NMR spectra were recorded at 25 ${}^{\circ }$ C using 5 mm NMR tubes and a Bruker 800 or 600 MHz NMR spectrometer equipped with TCI cryoprobes.

The isotope labelling efficiency of leucine residues in ubiquitin was assessed by integrating the ${}^{1}$ H-NMR signals of the $\delta_{2}$ -methyl group of Leu50 and its ${}^{13}$ C satellites, which are resolved in the 1D NMR spectrum. For samples without isotope-labelled leucine, the ${}^{13}$ C-HSQC cross-peak intensities of the labelled residues were compared with those of an internal standard of 0.1 mM 3- ${}^{13}$ C-pyruvate.

## 3 Results

### 3.1 Ubiquitin with ${}^{13}$ C-labelled methyl groups in alanine, leucine and valine made from 3- ${}^{13}$ C-pyruvate

**Figure 2:**
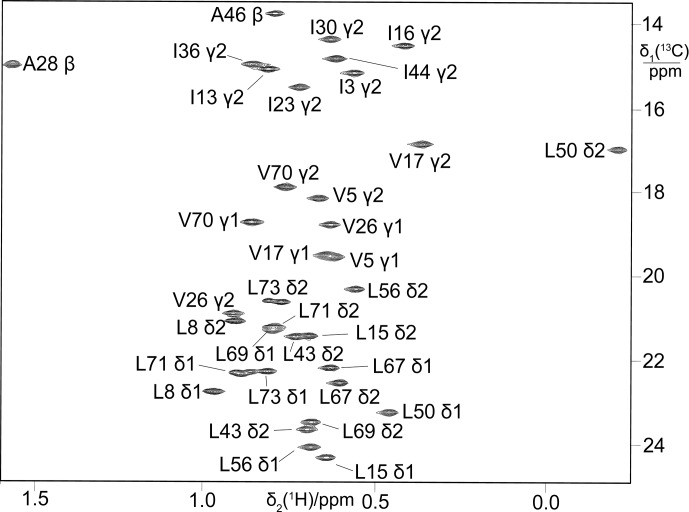
${}^{13}$ C-HSQC spectrum of ubiquitin produced from 3- ${}^{13}$ C-pyruvate by eCell CFPS, resulting in uniform ${}^{13}$ C labelling of both isopropyl methyl groups of leucine and valine. The protein yield was 0.7 mg from 10 mL eCell CFPS reaction, and the level of isotope labelling was 70 %.

The biosynthetic methyl labelling strategies were validated using ubiquitin as a model protein. The ${}^{13}$ C label was provided by 3- ${}^{13}$ C-pyruvate, which served both as carbon source for amino acid synthesis and an energy source for protein production. Omission of leucine and valine from the reaction mixture allows for the detection of ${}^{13}$ C-labelled valine and leucine produced from pyruvate during the cell-free reaction. As singly ${}^{13}$ C-labelled pyruvate contains no neighbouring ${}^{13}$ C atoms, the methyl groups of leucines and valines are expected to not show any ${}^{1}J_{\mathrm{CC}}$ coupling. This expectation was borne out in the experiment, where the cross-peaks revealed no splittings in the ${}^{13}$ C dimension (Fig. 2). Therefore, this labelling scheme delivers better spectral resolution than uniform ${}^{13}$ C-labelling schemes, where multiplet splittings due to ${}^{1}J_{\mathrm{CC}}$ couplings can be avoided only by specific pulse sequences that compromise sensitivity (Vuister and Bax, 1992; Behera et al., 2022). As the biosynthetic pathways remained intact, the ${}^{13}$ C label was subject to incorporation into a range of amino acids and thus prone to some isotope scrambling. For example, isotopic enrichment was also detected for alanine (due to direct conversion of pyruvate to alanine by alanine transaminase) and the $\gamma_{2}$ -methyl group of isoleucine. The labelling efficiency of the isopropyl groups of leucine and valine was about 70 %.

The absence of ${}^{13}$ C enrichment of the δ -methyl group of isoleucine is a signature of the biosynthetic pathway, where one pyruvate molecule is linked with unlabelled acetyl-CoA to form α -ketobutyrate as the precursor of isoleucine, channelling the ${}^{13}$ C label into the $\gamma_{2}$ -methyl rather than the δ -methyl group.

As the cell-free reaction was performed in a buffer containing high concentrations of glutamate, we speculated that the degree of isotope labelling could be increased by substituting glutamic acid for adipic acid, which is not easily converted into amino acids (Jia et al., 2009). Using 100 mM adipic acid and 8 mM MgCl ${}_{2}$ instead of 60 mM K-Glu and 8 mM Mg-Glu, however, did not increase the labelling efficiency and slightly decreased the protein yield (data not shown).

**Figure 3:**
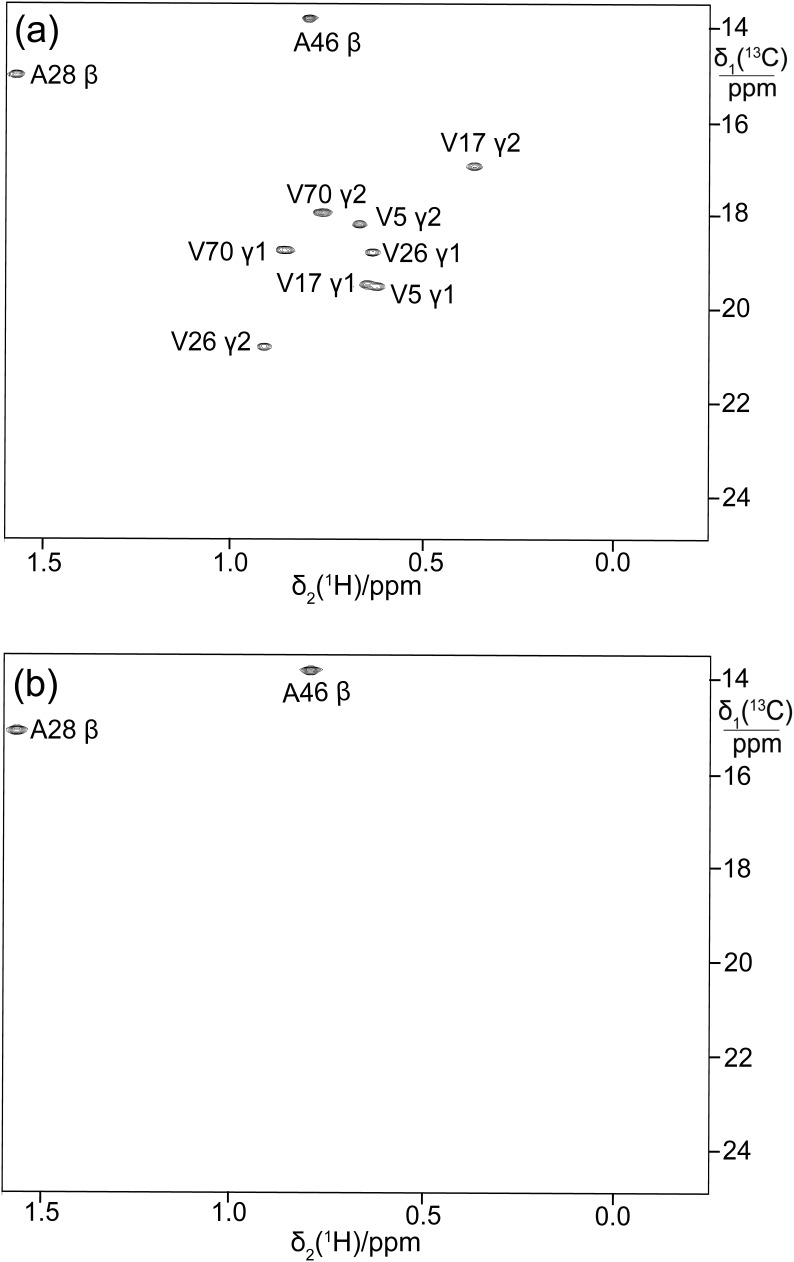
${}^{13}$ C-HSQC spectra of ubiquitin expressed in a 5 mL eCell CFPS reaction using 300 mg eCells with 3- ${}^{13}$ C-pyruvate. **(a)** Valine was omitted from the amino acid mixture. Protein yield 1.6 mg; ${}^{13}$ C enrichment of the valine methyl groups > 70 %. **(b)** Alanine was omitted from the amino acid mixture. Protein yield 1.35 mg; ${}^{13}$ C enrichment of the alanine methyl groups > 85 %.

Starting from 3- ${}^{13}$ C-pyruvate for biosynthesis, the selectivity of isotope labelling was enhanced by “unlabelling” the amino acids not of interest for labelling, which is achieved simply by adding them to the eCell CFPS reaction at natural isotopic abundance. For example, the ${}^{13}$ C label was apparent only in the valine methyl groups when only valine was omitted from the amino acid mixture (Fig. 3a), and only alanine peaks were observed when only alanine was left out (Fig. 3b).

### 3.2 Ubiquitin with ${}^{13}$ C-labelled methyl groups in leucine and valine made from 2- ${}^{13}$ C-methyl-acetolactate

It has been shown that 2- ${}^{13}$ C-methyl-acetolactate allows the in vivo production of proteins with stereospecifically labelled isopropyl groups of valine and leucine (Gans et al., 2010). To test the performance of this approach with eCells, a sample of ubiquitin was prepared with the provision of 2- ${}^{13}$ C-methyl-acetolactate and penoxsulam, which is a bactericidal acetolactate synthase (ALS) inhibitor that blocks the biosynthetic conversion of pyruvate to acetolactate, thus abolishing the synthesis of leucine and valine from pyruvate. Both the unlabelled pyruvate and creatine phosphate ATP regeneration systems were used. Both resulted in stereoselective labelling with similar labelling efficiency, highlighting the absence of any significant isotopic dilution by the addition of pyruvate at natural isotopic abundance. Figure 5 shows that the prochiral S -methyl groups of ubiquitin were stereoselectively labelled as expected. Although the ALS inhibitor did not entirely prevent the incorporation of unlabelled valine and leucine, presumably due to the unlabelled amino acids already present in the eCells prior to protein production, the isotope labelling efficiency nevertheless reached 70 %. Importantly, the eCell system enabled the production of this selectively ${}^{13}$ C-labelled sample from less than 6 mg methyl-acetolactate precursor, and no ${}^{13}$ C labelling of pro- R methyl groups was detectable. The effectiveness of the ALS inhibitor in preventing the production of unlabelled valine and leucine was confirmed by comparison with the isotope labelling efficiency when the eCell CFPS was performed using the widely used ATP regeneration system with creatine phosphate and creatine kinase (Kigawa et al., 1999; Apponyi et al., 2008). The same isotope labelling efficiency and the same protein yield (0.7 mg) were obtained.

**Figure 4:**
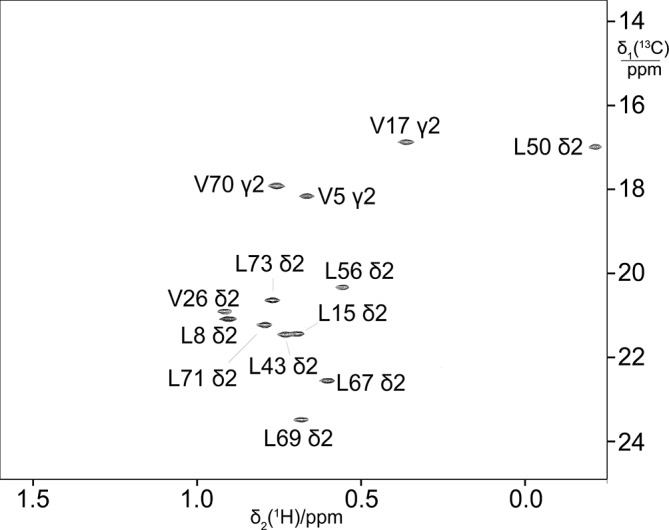
${}^{13}$ C-HSQC spectrum of ubiquitin with labelling of the pro- S methyl groups in leucine and valine by using site-specifically ${}^{13}$ C-labelled acetolactate in eCell CFPS. Protein yield 0.7 mg, isotope labelling efficiency 70 %.

### 3.3 PpiB with stereospecific ${}^{13}$ C-labelled methyl groups in valine

**Figure 5:**
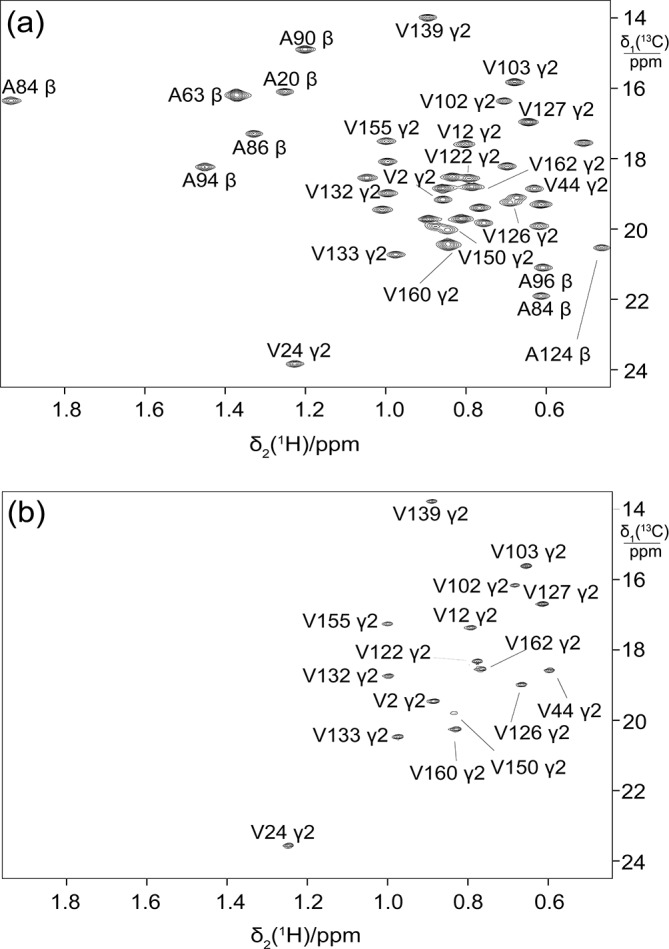
Selective ${}^{13}$ C labelling of the methyl groups of alanine and valine residues in PpiB produced by eCell CFPS. **(a)** ${}^{13}$ C-HSQC spectrum of PpiB produced from 3- ${}^{13}$ C-pyruvate with valine omitted. Published assignments are shown (BMRB file 11451). The spectrum also displays the cross-peaks of the $\gamma_{1}$ -methyl groups, but their assignments have not been reported. Protein yield 2.2 mg; isotope labelling efficiency > 75 %. **(b)** ${}^{13}$ C-HSQC spectrum of PpiB produced from 2- ${}^{13}$ C-methyl-acetolactate by eCell CFPS with valine omitted in an eCell CFPS reaction in D ${}_{2}$ O using deuterated eCells. The ${}^{13}$ C-HSQC spectrum illustrates the selective labelling of the pro- S -methyl groups of valine in a perdeuterated protein. The protein yield was 1.3 mg, and the ${}^{13}$ C-labelling level was 90 %.

To illustrate the broad applicability of the eCell approach to produce perdeuterated proteins, it was also applied to the *E. coli* peptidyl-prolyl *cis–trans* isomerase B (PpiB), which is a 19 kDa protein. Figure 5a shows the ${}^{13}$ C-HSQC cross-peaks of PpiB prepared with 3- ${}^{13}$ C-pyruvate while omitting valine. Although the methyl groups of alanine residues are also observed, no two cross-peaks overlap to the extent that they cannot be recognized as separate cross-peaks.

Figure 5b shows the ${}^{13}$ C-HSQC cross-peaks of perdeuterated PpiB made by eCell CFPS using perdeuterated eCells and 2- ${}^{13}$ C-methyl-4- ${}^{2}$ H ${}_{3}$ -acetolactate. All amino acids were provided in perdeuterated form and valine was omitted. This resulted in stereoselective labelling of the pro- S groups of valine residues in PpiB with a high labelling efficiency (ca. 90 %) and adequate yield (1.32 mg). The deuteration level of the protein was high, as shown by a 1D ${}^{1}$ H-NMR spectrum (Fig. S3 in the Supplement).

### 3.4 eCell CFPS for stereospecific assignments by biosynthetically directed fractional ${}^{13}$ C labelling

**Figure 6:**
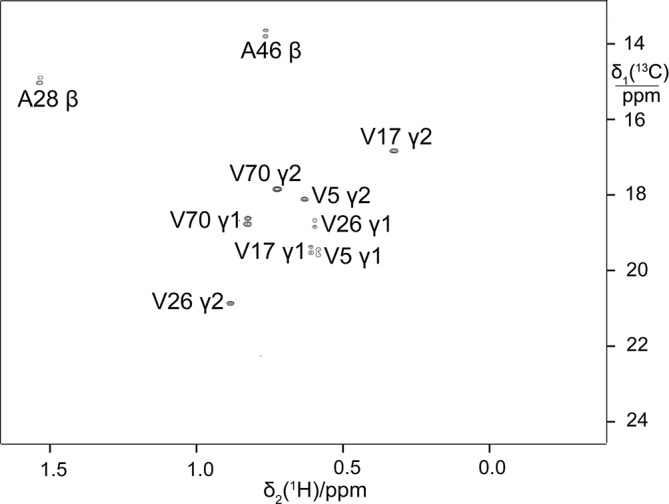
${}^{13}$ C-HSQC spectrum of ubiquitin produced by eCell CFPS from a mixture of 10 % uniformly ${}^{13}$ C-labelled glucose and 90 % glucose at natural isotopic abundance. The sample was prepared using 300 mg eCells in 5 mL CFPS buffer with 3 mg ${}^{13}$ C-glucose and 27 mg unlabelled glucose. Protein yield 3.8 mg; labelling efficiency below 10 %.

Biosynthetic fractional ${}^{13}$ C labelling is a well-established approach to obtain stereospecific assignments of isopropyl methyl groups (Senn et al., 1989; Neri et al., 1989; Schubert et al., 2006). Starting from a mixture of 10 % uniformly ${}^{13}$ C-labelled glucose and 90 % glucose at natural isotopic abundance, the ${}^{13}$ C-NMR spectrum of pro- R methyl groups displays splittings due to ${}^{1}J_{\mathrm{CC}}$ couplings while the pro- S methyl groups do not. The approach is inexpensive as only little isotope-labelled glucose is needed. To explore whether eCells maintain the required biosynthetic pathway, a sample of ubiquitin was prepared from a mixture of ${}^{13}$ C-labelled and unlabelled glucose. The ${}^{13}$ C-HSQC spectrum showed the multiplet fine structures expected for the pro- R and pro- S methyl groups (Fig. 6).

## 4 Discussion

The present work shows that biosynthetic pathways naturally established in bacterial cells can be exploited to produce selectively ${}^{13}$ C-labelled proteins also in a cell-free reaction to deliver protein in yields sufficient for NMR analysis. In contrast to the preparation of cell extracts by mechanical lysis and high-speed centrifugation, the preparation of eCells uses milder conditions and thus stands a greater chance of preserving the activities of the natural complement of biosynthetic enzymes of the parent live *E. coli* cells. In this way, eCells combine intact biosynthetic pathways with some of the fundamental advantages of conventional CFPS, namely the low requirement of amino acids (Torizawa et al., 2004), greater likelihood of compatibility with toxic proteins and facile modification of the solution conditions with regard to compounds small enough to enter the eCells. Importantly, eCells can be produced rapidly and easily. Once prepared, they can be stored at - 80 ${}^{\circ }$ C for years without loss of activity.

The present work explored the activity of biosynthetic enzymes towards valine and leucine in eCells. As anticipated for preserved biosynthetic pathways, we readily obtained protein samples with ${}^{13}$ CH ${}_{3}$ -labelled valine and leucine, where the amino acids were made from inexpensive pyruvate during the eCell CFPS reaction. Starting from 3- ${}^{13}$ C-pyruvate, the scheme maximizes the spectral resolution of the ${}^{13}$ C-HSQC cross-peaks of different methyl groups by avoiding multiplet splittings arising from large ${}^{1}J_{\mathrm{CC}}$ coupling constants. As the biosynthetic pathway from pyruvate to valine and leucine appears intact, it was not surprising to also observe facile conversion of 2- ${}^{13}$ C-methyl-4-acetolactate to valine.

Furthermore, the eCell system proved capable of converting glucose into alanine, valine and leucine, allowing the stereospecific distinction of the isopropyl methyl groups by the classical method of biosynthetic fractional ${}^{13}$ C labelling that uses an inexpensive mixture of uniformly ${}^{13}$ C-labelled glucose with an excess of glucose at natural isotopic abundance (Neri et al., 1989). Biosynthetic fractional ${}^{13}$ C labelling in eCell CFPS allows for stereospecific assignments at extraordinarily low cost as far as ${}^{13}$ C-labelled glucose is concerned, but the level of isotope labelling associated with this scheme is intrinsically low, and we therefore prefer 2- ${}^{13}$ C-methyl-4-acetolactate for stereospecific assignments, which also minimizes cross-peak overlap by avoiding ${}^{1}J_{\mathrm{CC}}$ multiplet splittings.

Stereospecific ${}^{13}$ C labelling with 2- ${}^{13}$ C-methyl-4-acetolactate in in vivo protein expression (Gans et al., 2010) has become very popular, and this precursor is available commercially. (We found the deuterated isotopologue 2- ${}^{13}$ C-methyl-4- ${}^{2}$ H ${}_{3}$ -acetolactate to be more readily available than the undeuterated analogue, although the selective ${}^{13}$ C-labelling strategy would be beneficial also without deuteration.) Our results show that eCell CFPS requires only small amounts of 2-methyl-acetolactate to produce proteins for identification of the pro- S methyl groups in ${}^{13}$ C-HSQC spectra. To use this labelling scheme in combination with perdeuteration, we supplied all other amino acids in perdeuterated form. While this increases the cost of isotope-labelled material, the labelling scheme is still affordable. Table 1 shows the cost for isotope-labelled precursors used in the experiments of the present work.

**Table 1:** Comparison of precursors and their contribution to the cost of eCell CFPS reaction with 300 mg eCells. ${}^{1}$

${}^{13}$ C-labelled precursor	Precursor cost	Cost of	Total	Labelling	Position
		precursor for	protein	( ${}^{\circ }$ )	labelled
		one reaction	yield		
			(mg mL ${}^{-1}$ )		
2- ${}^{13}$ C-methyl-4- ${}^{2}$ H ${}_{3}$ -acetolactate	USD 1722 g ${}^{-1}$	USD 14 ${}^{2}$	0.7	90 %	$V=\gamma_{2}$
					$L=\delta_{2}$
3- ${}^{13}$ C-pyruvate	USD 866 g ${}^{-1}$	USD 34	0.8	70 %	$V=\gamma_{2}$ , $\gamma_{1}$
					$L=\delta_{2}$ , $\delta_{1}$
					$I=\gamma_{2}$
10 % [U- ${}^{13}$ C]-glucose +90 %	USD 258 g ${}^{-1}$	USD 2	3.8	10 %	$V=\gamma_{2}^{3}$
unlabelled glucose					
1- ${}^{13}$ C-glucose	USD 282 g ${}^{-1}$	USD 14	1.4	44 %	$V=\gamma_{2}$ , $\gamma_{1}$
					$L=\delta_{2}$ , $\delta_{1}$

${}^{1}$ Prices from Cambridge Isotope Laboratories (https://www.isotope.com, last access: 3 April 2023), Omicron Biochemicals Inc. (https://www.omicronbio.com, last access: 3 April 2023) and Apollo Scientific (D ${}_{2}$ O; https://store.apolloscientific.co.uk, last access: 3 April 2023). ${}^{2}$ Additional isotope costs were for perdeuterated amino acids (USD 260; Table S1) and the D ${}_{2}$ O (USD 838 per litre; USD 377 for the 0.45 L cell culture used) for growing perdeuterated *E. coli* cells. ${}^{3}$ eCells can synthesize leucine from glucose (Fig. S2). Therefore, stereospecific isotope labelling of the $\delta_{2}$ position of leucine may also be achieved, but this was not tested experimentally.

Purified ILV amino acids with stereospecific ${}^{13}$ C enrichment of single methyl groups are commercially available but expensive. As an alternative, Linser et al. (2014) showed that CFPS reactions can be conducted with an amino acid mixture produced by hydrolysis of a suitably isotope-labelled protein expressed in vivo. Also in this approach, however, some amino acids need to be supplied in purified form if they are degraded during hydrolysis of the labelled protein. Assembling the amino acid mixture from commercially available individual components is less laborious and offers the important advantage that a single amino acid can be omitted and thus targeted for production by biosynthesis. In this way we obtained high levels of ${}^{13}$ C incorporation (90 %) and deuteration (estimated to be > 95 %), which are comparable with in vivo protein preparations and favourable for good sensitivity of NMR experiments of large protein complexes (O'Brien et al., 2018). In practice, the economical use of amino acids in the eCell CFPS reaction meant that the cost of D ${}_{2}$ O used for producing perdeuterated eCells (USD 377) exceeded that of the perdeuterated amino acids added in the CFPS reaction (USD 260, Table S1).

Pyruvate plays a central role in bacterial biosynthesis, and, as shown in the present work, singly ${}^{13}$ C-labelled pyruvate is suitable as a relatively inexpensive precursor for labelling methyl groups of leucine and valine with high levels of ${}^{13}$ C enrichment. If, at the same time, unlabelled leucine or valine is provided in the CFPS reaction to suppress their respective cross-peaks, the cross-peaks of the amino acid omitted can be observed selectively. The increased spectral resolution afforded by this scheme is particularly beneficial for larger proteins. Furthermore, inactivation of transaminases by reduction with NaBH ${}_{4}$ (Su et al., 2011) may allow extending this approach to the selective ${}^{15}$ N labelling of amino acids from ${}^{15}$ N-ammonium salt. These experiments are currently in progress.

In principle, using 1- ${}^{13}$ C-glucose as the carbon source delivers the same selectivity of isotope labelling as 3- ${}^{13}$ C-pyruvate (Lundström et al., 2007), but, as glycolysis breaks the glucose down into 3- ${}^{13}$ C-pyruvate and unlabelled pyruvate, glucose simultaneously labelled in the 1 and 6 position is required to avoid the dilution with unlabelled pyruvate (Loquet et al., 2011). We therefore prefer 3- ${}^{13}$ C-pyruvate.

As pyruvate can be converted to alanine by a single enzyme, it is difficult to suppress the cross-peaks of the C ${}^{\beta }$ H ${}_{3}$ groups of alanine when starting from ${}^{13}$ C-labelled pyruvate. The addition of an excess of unlabelled alanine to the reaction would dilute the labelled pyruvate with unlabelled pyruvate, and inhibition of the alanine aminotransferase by reduction with NaBH ${}_{4}$ would also inhibit the transaminase that instals the amino group on leucine and valine by transfer from glutamate. We therefore propose to identify the alanine cross-peaks with a sample, where the isotope labelling of leucine and valine is suppressed by the provision of these amino acids in unlabelled form (Fig. 3b).

Starting from pyruvate, we found it difficult to achieve ${}^{13}$ C-labelling efficiencies much above 70 %. We attribute this to an isotope dilution effect due to a pool of unlabelled amino acids present in the eCells. Attempts to dialyse eCells in a large volume of buffer for an extended period of time reduced the protein yield as the eCells lose activity by gradually leaking bio-macromolecules (Van Raad and Huber, 2021). Notably, proteins produced in vivo from various ${}^{13}$ C-labelled glucose isotopomers are likewise subject to isotopic dilution, and examples with ∼ 45 % labelling efficiency have been reported (Lundström et al., 2009; Loquet et al., 2011; Weininger, 2017).

As proteins slowly leak through the porous polymer coating, the lifetime of eCells is limited to about 8 h at 37 ${}^{\circ }$ C, which limits protein yields. We note, however, that it is recommended not to conduct in vivo protein expression from selectively labelled precursors for too long either to avoid isotope scrambling by precursor recycling (Kurauskas et al., 2017).

## 5 Conclusions

In summary, the eCell platform opens new possibilities for the selective ${}^{13}$ C enrichment of methyl groups in proteins. It combines high levels of isotope enrichment with low cost of isotope-enriched precursors. The protocol for eCell preparation is uncomplicated, and the ready accessibility of the interior space of eCells to low molecular-weight compounds provides control over the chemical environment, so that different isotope labelling is achieved simply by the use of different reaction buffers. In contrast to conventional CFPS based on dialysis systems, where protein yields depend on good contact between inside and outside buffers and, therefore, the geometry of the setup, the eCell system can readily be scaled in volume. We anticipate that it will find many more uses beyond those demonstrated in the present work.

## Supplement

10.5194/mr-4-187-2023-supplement The supplement contains the nucleotide sequences of genes used in this work and the high-resolution mass spectrum of the deuterated PpiB sample. The supplement related to this article is available online at: https://doi.org/10.5194/mr-4-187-2023-supplement.

## Data Availability

The NMR data are available at https://doi.org/10.5281/zenodo.7662927 (Van Raad et al., 2023).
