# Supplementary material for: Cell-free synthesis of proteins with selectively 13C-labelled methyl groups from inexpensive precursors
Source: Magn Reson (Gott). 2023 Jul 19;4(2):187–97. doi: 10.5194/mr-4-187-2023 (PMC10583297; doi:10.5194/mr-4-187-2023)
Supplement: The supplement contains the nucleotide sequences of genes used in this work and the high-resolution mass spectrum of the deuterated PpiB sample. The supplement related to this article is available online at: https://doi.org/10.5194/mr-4-187-2023-supplement. [file mr-4-187-supplement.pdf]

Supplement of Magn. Reson., 4, 187–197, 2023
https://doi.org/10.5194/mr-4-187-2023-supplement
© Author(s) 2023. CC BY 4.0 License.

Open Access
MAGNETIC RESONANCE

Supplement of

Cell-free synthesis of proteins with selectively ^{13}C-labelled methyl groups from inexpensive precursors

Damian Van Raad et al.

Correspondence to: Gottfried Otting (gottfried.otting@anu.edu.au) and Thomas Huber (t.huber@anu.edu.au)

The copyright of individual parts of the supplement might differ from the article licence.

Supporting Information

Nucleotide sequences

pCDF CTH lac PpiB

AGATCTCGATCCCGCGAAATTAATACGACTCACTATAGGGGAATTGTGAGCGGATAACAATTCCGAGACCACA
ACGGTTTCCCTCTAGAAATAATTTTGTTTAACTTTAAGAAGGAGATATACATATGGTTACTTTCCACACCAATCA
CGGCGATATTGTCATCAAAACTTTTGACGATAAAGCACCTGAAACAGTTAAAAACTTCCTGGACTACTGCCGC
GAAGGTTTTTACAACAACACCATTTTCCACCGTGTTATCAACGGCTTTATGATTCAGGGCGGCGGTTTTGAACC
GGGCATGAAACAAAAAGCCACCAAAGAACCGATCAAAAACGAAGCCAACAACGGCCTGAAAAATACCCGTG
GTACGCTGGCAATGGCACGTACTCAGGCTCCGCACTCTGCAACTGCACAGTTCTTCATCAACGTGGTTGATAA
CGACTTCCTGAACTTCTCTGGCGAAAGCCTGCAAGGTTGGGGCTACTGCGTGTTTGCTGAAGTGGTTGACGGC
ATGGACGTGGTAGACAAAATCAAAGGTGTTGCAACCGGTCGTAGCGGTATGCATCAGGACGTGCCAAAAGA
AGACGTTATCATTGAAAGCGTGACCGTTAGCGAGCACCACCATCATCACCACTAAGAATTCGAGCTCCCGGGT
ACCATGGCATGCATCGATAGATCCGGCTGCTAACAAAGCCCGAAAGGAAGCTGAGTTGGCTGCTGCCACCGC
TGAGCAATAACTAGCATAACCCCTTGGGGCCTCTAAACGGGTCTTGAGGGGTTTTTTGCTGAAAGGAGGAACT
ACAGGCATTTGAGAAGCACACGGTCACACTGCTTCCGGTAGTCAATAAACCGGTAAACCAGCAATAGACATAA
GCGGCTATTTAACGACCCTGCCCTGAACCGACGACCGGGTCATCGTGGCCGGATCTTGCGGCCCCTCGGCTTG
AACGAATTGTTAGACATTATTTGCCGACTACCTTGGTGATCTCGCCTTTCACGTAGTGGACAAATTCTTCCAACT
GATCTGCGCGCGAGGCCAAGCGATCTTCTTCTTGTCCAAGATAAGCCTGTCTAGCTTCAAGTATGACGGGCTG
ATACTGGGCCGGCAGGCGCTCCATTGCCCAGTCGGCAGCGACATCCTTCGGCGCGATTTTGCCGGTTACTGCG
CTGTACCAAATGCGGGACAACGTAAGCACTACATTTCGCTCATCGCCAGCCCAGTCGGGCGGCGAGTTCCATA
GCGTTAAGGTTTCATTTAGCGCCTCAAATAGATCCTGTTCAGGAACCGGATCAAAGAGTTCCTCCGCCGCTGG
ACCTACCAAGGCAACGCTATGTTCTCTTGCTTTTGTCAGCAAGATAGCCAGATCAATGTCGATCGTGGCTGGCT
CGAAGATACCTGCAAGAATGTCATTGCGCTGCCATTCTCCAAATTGCAGTTCGCGCTTAGCTGGATAACGCCA
CGGAATGATGTCGTCGTGCACAACAATGGTGACTTCTACAGCGCGGAGAATCTCGCTCTCTCCAGGGGAAGC
CGAAGTTTCCAAAAGGTCGTTGATCAAAGCTCGCCGCGTTGTTTCATCAAGCCTTACGGTCACCGTAACCAGC
AAATCAATATCACTGTGTGGCTTCAGGCCGCCATCCACTGCGGAGCCGTACAAATGTACGGCCAGCAACGTCG
GTTCGAGATGGCGCTCGATGACGCCAACTACCTCTGATAGTTGAGTCGATACTTCGGCGATCACCGCTTCCCTC
ATACTCTTCCTTTTTCAATATTATTGAAGCATTTATCAGGGTTATTGTCTCATGAGCGGATACATATTTGAATGT
ATTTAGAAAAATAAACAAATAGCTAGCTCACTCGGTCGCTACGCTCCGGGCGTGAGACTGCGGCGGGCGCTG
CGGACACATACAAAGTTACCCACAGATTCCGTGGATAAGCAGGGGACTAACATGTGAGGCAAAACAGCAGG
GCCGCGCCGGTGGCGTTTTTCCATAGGCTCCGCCCTCCTGCCAGAGTTCACATAAACAGACGCTTTTCCGGTGC
ATCTGTGGGAGCCGTGAGGCTCAACCATGAATCTGACAGTACGGGCGAAACCCGACAGGACTTAAAGATCCC
CACCGTTTCCGGCGGGTCGCTCCCTCTTGCGCTCTCCTGTTCCGACCCTGCCGTTTACCGGATACCTGTTCCGCC
TTTCTCCCTTACGGGAAGTGTGGCGCTTTCTCATAGCTCACACACTGGTATCTCGGCTCGGTGTAGGTCGTTCG
CTCCAAGCTGGGCTGTAAGCAAGAACTCCCCGTTCAGCCCGACTGCTGCGCCTTATCCGGTAACTGTTCACTTG
AGTCCAACCCGGAAAAGCACGGTAAAACGCCACTGGCAGCAGCCATTGGTAACTGGGAGTTCGCAGAGGATT
TGTTTAGCTAAACACGCGGTTGCTCTTGAAGTGTGCGCCAAAGTCCGGCTACACTGGAAGGACAGATTTGGTT
GCTGTGCTCTGCGAAAGCCAGTTACCACGGTTAAGCAGTTCCCCAACTGACTTAACCTTCGATCAAACCACCTC
CCCAGGTGGTTTTTTCGTTTACAGGGCAAAAGATTACGCGCAGAAAAAAAGGATCTCAAGAAGATCCTTTGAT
CTTTTCTACTGAACCGCTCTAGATTTCAGTGCAATTTATCTCTTCAAATGTAGCACCTGAAGTCAGCCCCATACG
ATATAAGTTGTAATTCTCATGTTAGTCATGCCCCGCGCCCACCGGAAGGAGCTGACTGGGTTGAAGGCTCTCA
AGGGCATCGGTCGAGATCCCGGTGCCTAATGAGTGAGCTAACTTACATTAATTGCGTTGCGCTCACTGCCCGC
TTTCCAGTCGGGAAACCTGTCGTGCCAGCTGCATTAATGAATCGGCCAACGCGCGGGGAGAGGCGGTTTGCG
TATTGGGCGCCAGGGTGGTTTTTCTTTTCACCAGTGAGACGGGCAACAGCTGATTGCCCTTCACCGCCTGGCC

CTGAGAGAGTTGCAGCAAGCGGTCCACGCTGGTTTGCCCCAGCAGGCGAAAATCCTGTTTGATGGTGGTTAA
CGGCGGGATATAACATGAGCTGTCTTCGGTATCGTCGTATCCCACTACCGAGATGTCCGCACCAACGCGCAGC
CCGGACTCGGTAATGGCGCGCATTGCGCCCAGCGCCATCTGATCGTTGGCAACCAGCATCGCAGTGGGAACG
ATGCCCTCATTCAGCATTTGCATGGTTTGTTGAAAACCGGACATGGCACTCCAGTCGCCTTCCCGTTCCGCTAT
CGGCTGAATTTGATTGCGAGTGAGATATTTATGCCAGCCAGCCAGACGCAGACGCGCCGAGACAGAACTTAA
TGGGCCCGCTAACAGCGCGATTTGCTGGTGACCCAATGCGACCAGATGCTCCACGCCCAGTCGCGTACCGTCT
TCATGGGAGAAAATAATACTGTTGATGGGTGTCTGGTCAGAGACATCAAGAAATAACGCCGGAACATTAGTG
CAGGCAGCTTCCACAGCAATGGCATCCTGGTCATCCAGCGGATAGTTAATGATCAGCCCACTGACGCGTTGCG
CGAGAAGATTGTGCACCGCCGCTTTACAGGCTTCGACGCCGCTTCGTTCTACCATCGACACCACCACGCTGGC
ACCCAGTTGATCGGCGCGAGATTTAATCGCCGCGACAATTTGCGACGGCGCGTGCAGGGCCAGACTGGAGGT
GGCAACGCCAATCAGCAACGACTGTTTGCCCGCCAGTTGTTGTGCCACGCGGTTGGGAATGTAATTCAGCTCC
GCCATCGCCGCTTCCACTTTTTCCCGCGTTTTCGCAGAAACGTGGCTGGCCTGGTTCACCACGCGGGAAACGG
TCTGATAAGAGACACCGGCATACTCTGCGACATCGTATAACGTTACTGGTTTCACATTCACCACCCTGAATTGA
CTCTCTTCCGGGCGCTATCATGCCATACCGCGAAAGGTTTTGCGCCATTCGATGGTGTCCGGGATCTCGACGCT
CTCCCTTATGCGACTCCTGCATTAGGTCGGGTTGTCAGCCTGTCCCGC

pCDF lac CTH ubi

AGATCTCGATCCCGCGAAATTAATACGACTCACTATAGGGGAATTGTGAGCGGATAACAATTCCCCTCTAGAA
ATAATTTTGTTTAACTTTAAGAAGGAGATATACATATGCAGATCTTCGTGAAGACTCTGACTGGTAAGACCATC
ACCCTCGAGGTTGAGCCCAGTGACACCATTGAGAATGTCAAGGCAAAGATCCAAGATAAGGAAGGCATCCCT
CCTGACCAGCAGAGGCTGATCTTTGCTGGAAAACAGCTGGAAGATGGGCGCACCCTGTCTGACTACAACATCC
AGAAAGAGTCCACCCTGCACCTGGTACTCCGTCTCAGAGGTGGATCTCATCACCATCACCATCACTAAGAATTC
GAGCTCCCGGGTACCATGGCATGCATCGATAGATCCGGCTGCTAACAAAGCCCGAAAGGAAGCTGAGTTGGC
TGCTGCCACCGCTGAGCAATAACTAGCATAACCCCTTGGGGCCTCTAAACGGGTCTTGAGGGGTTTTTTGCTG
AAAGGAGGAACTACAGGCATTTGAGAAGCACACGGTCACACTGCTTCCGGTAGTCAATAAACCGGTAAACCA
GCAATAGACATAAGCGGCTATTTAACGACCCTGCCCTGAACCGACGACCGGGTCATCGTGGCCGGATCTTGCG
GCCCCTCGGCTTGAACGAATTGTTAGACATTATTTGCCGACTACCTTGGTGATCTCGCCTTTCACGTAGTGGAC
AAATTCTTCCAACTGATCTGCGCGCGAGGCCAAGCGATCTTCTTCTTGTCCAAGATAAGCCTGTCTAGCTTCAA
GTATGACGGGCTGATACTGGGCCGGCAGGCGCTCCATTGCCCAGTCGGCAGCGACATCCTTCGGCGCGATTT
TGCCGGTTACTGCGCTGTACCAAATGCGGGACAACGTAAGCACTACATTTCGCTCATCGCCAGCCCAGTCGGG
CGGCGAGTTCCATAGCGTTAAGGTTTCATTTAGCGCCTCAAATAGATCCTGTTCAGGAACCGGATCAAAGAGT
TCCTCCGCCGCTGGACCTACCAAGGCAACGCTATGTTCTCTTGCTTTTGTCAGCAAGATAGCCAGATCAATGTC
GATCGTGGCTGGCTCGAAGATACCTGCAAGAATGTCATTGCGCTGCCATTCTCCAAATTGCAGTTCGCGCTTA
GCTGGATAACGCCACGGAATGATGTCGTCGTGCACAACAATGGTGACTTCTACAGCGCGGAGAATCTCGCTCT
CTCCAGGGGAAGCCGAAGTTTCCAAAAGGTCGTTGATCAAAGCTCGCCGCGTTGTTTCATCAAGCCTTACGGT
CACCGTAACCAGCAAATCAATATCACTGTGTGGCTTCAGGCCGCCATCCACTGCGGAGCCGTACAAATGTACG
GCCAGCAACGTCGGTTCGAGATGGCGCTCGATGACGCCAACTACCTCTGATAGTTGAGTCGATACTTCGGCGA
TCACCGCTTCCCTCATACTCTTCCTTTTTCAATATTATTGAAGCATTTATCAGGGTTATTGTCTCATGAGCGGATA
CATATTTGAATGTATTTAGAAAAATAAACAAATAGCTAGCTCACTCGGTCGCTACGCTCCGGGCGTGAGACTG
CGGCGGGCGCTGCGGACACATACAAAGTTACCCACAGATTCCGTGGATAAGCAGGGGACTAACATGTGAGG
CAAAACAGCAGGGCCGCGCCGGTGGCGTTTTTCCATAGGCTCCGCCCTCCTGCCAGAGTTCACATAAACAGAC
GCTTTTCCGGTGCATCTGTGGGAGCCGTGAGGCTCAACCATGAATCTGACAGTACGGGCGAAACCCGACAGG
ACTTAAAGATCCCCACCGTTTCCGGCGGGTCGCTCCCTCTTGCGCTCTCCTGTTCCGACCCTGCCGTTTACCGG
ATACCTGTTCCGCCTTTCTCCCTTACGGGAAGTGTGGCGCTTTCTCATAGCTCACACACTGGTATCTCGGCTCG
GTGTAGGTCGTTCGCTCCAAGCTGGGCTGTAAGCAAGAACTCCCCGTTCAGCCCGACTGCTGCGCCTTATCCG
GTAACTGTTCACTTGAGTCCAACCCGGAAAAGCACGGTAAAACGCCACTGGCAGCAGCCATTGGTAACTGGG

AGTTCGCAGAGGATTTGTTTAGCTAAACACGCGGTTGCTCTTGAAGTGTGCGCCAAAGTCCGGCTACACTGGA
AGGACAGATTTGGTTGCTGTGCTCTGCGAAAGCCAGTTACCACGGTTAAGCAGTTCCCCAACTGACTTAACCTT
CGATCAAACCACCTCCCCAGGTGGTTTTTTCGTTTACAGGGCAAAAGATTACGCGCAGAAAAAAAGGATCTCA
AGAAGATCCTTTGATCTTTTCTACTGAACCGCTCTAGATTTCAGTGCAATTTATCTCTTCAAATGTAGCACCTGA
AGTCAGCCCCATACGATATAAGTTGTAATTCTCATGTTAGTCATGCCCCGCGCCCACCGGAAGGAGCTGACTG
GGTTGAAGGCTCTCAAGGGCATCGGTCGAGATCCCGGTGCCTAATGAGTGAGCTAACTTACATTAATTGCGTT
GCGCTCACTGCCCGCTTTCCAGTCGGGAAACCTGTCGTGCCAGCTGCATTAATGAATCGGCCAACGCGCGGGG
AGAGGCGGTTTGCGTATTGGGCGCCAGGGTGGTTTTTCTTTTCACCAGTGAGACGGGCAACAGCTGATTGCCC
TTCACCGCCTGGCCCTGAGAGAGTTGCAGCAAGCGGTCCACGCTGGTTTGCCCCAGCAGGCGAAAATCCTGTT
TGATGGTGGTTAACGGCGGGATATAACATGAGCTGTCTTCGGTATCGTCGTATCCCACTACCGAGATGTCCGC
ACCAACGCGCAGCCCGGACTCGGTAATGGCGCGCATTGCGCCCAGCGCCATCTGATCGTTGGCAACCAGCAT
CGCAGTGGGAACGATGCCCTCATTCAGCATTTGCATGGTTTGTTGAAAACCGGACATGGCACTCCAGTCGCCT
TCCCGTTCCGCTATCGGCTGAATTTGATTGCGAGTGAGATATTTATGCCAGCCAGCCAGACGCAGACGCGCCG
AGACAGAACTTAATGGGCCCGCTAACAGCGCGATTTGCTGGTGACCCAATGCGACCAGATGCTCCACGCCCA
GTCGCGTACCGTCTTCATGGGAGAAAATAATACTGTTGATGGGTGTCTGGTCAGAGACATCAAGAAATAACG
CCGGAACATTAGTGCAGGCAGCTTCCACAGCAATGGCATCCTGGTCATCCAGCGGATAGTTAATGATCAGCCC
ACTGACGCGTTGCGCGAGAAGATTGTGCACCGCCGCTTTACAGGCTTCGACGCCGCTTCGTTCTACCATCGAC
ACCACCACGCTGGCACCCAGTTGATCGGCGCGAGATTTAATCGCCGCGACAATTTGCGACGGCGCGTGCAGG
GCCAGACTGGAGGTGGCAACGCCAATCAGCAACGACTGTTTGCCCGCCAGTTGTTGTGCCACGCGGTTGGGA
ATGTAATTCAGCTCCGCCATCGCCGCTTCCACTTTTTCCCGCGTTTTCGCAGAAACGTGGCTGGCCTGGTTCAC
CACGCGGGAAACGGTCTGATAAGAGACACCGGCATACTCTGCGACATCGTATAACGTTACTGGTTTCACATTC
ACCACCCTGAATTGACTCTCTTCCGGGCGCTATCATGCCATACCGCGAAAGGTTTTGCGCCATTCGATGGTGTC
CGGGATCTCGACGCTCTCCCTTATGCGACTCCTGCATTAGGTCGGGTTGTCAGCCTGTCCCGC

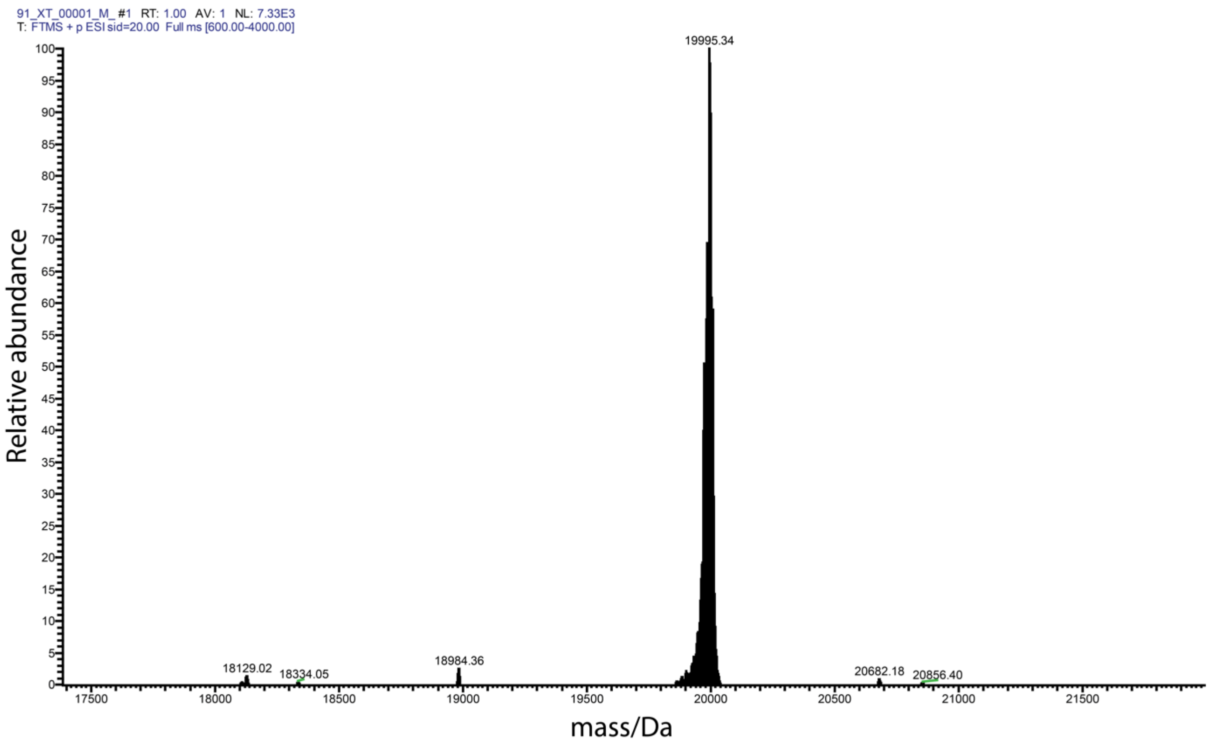

Figure S1. High-resolution mass spectrum of perdeuterated PpiB. The calculated mass for complete deuteration is 20,257 Da (18,966 Da at natural isotopic abundance, including the mass of the C-terminal His_6-tag of the construct). The perdeuterated amino acids lysine, arginine, histidine and glutamine added to the reaction mixture also contained ^{15}N, whereas all other non-valine amino acids were perdeuterated without any other heavy isotope. The mass observed indicates > 90% deuteration of PpiB, taking into account the back-exchange of exchangeable protons during protein purification.

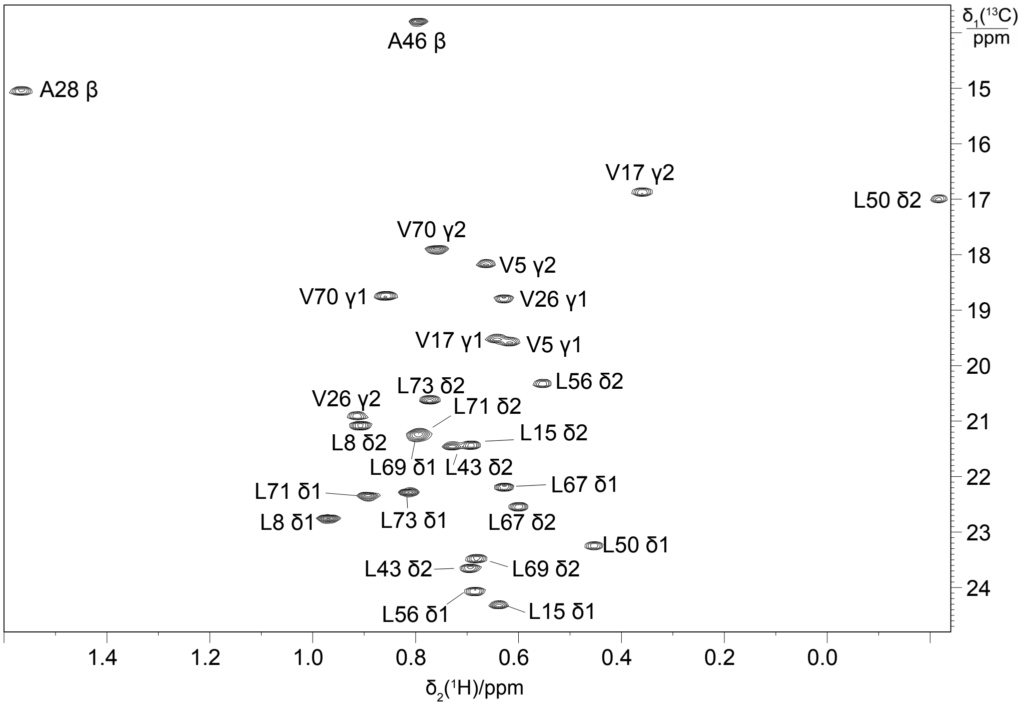

Figure S2. ^{13}C-HSQC spectra of ubiquitin expressed in a 5 mL CFPS reaction using 300 mg eCells with 1-^{13}C-glucose. Valine and leucine were omitted from the amino acid mixture. Protein yield 1.4 mg, ^{13}C-enrichment of the valine/leucine methyl groups ~44%.

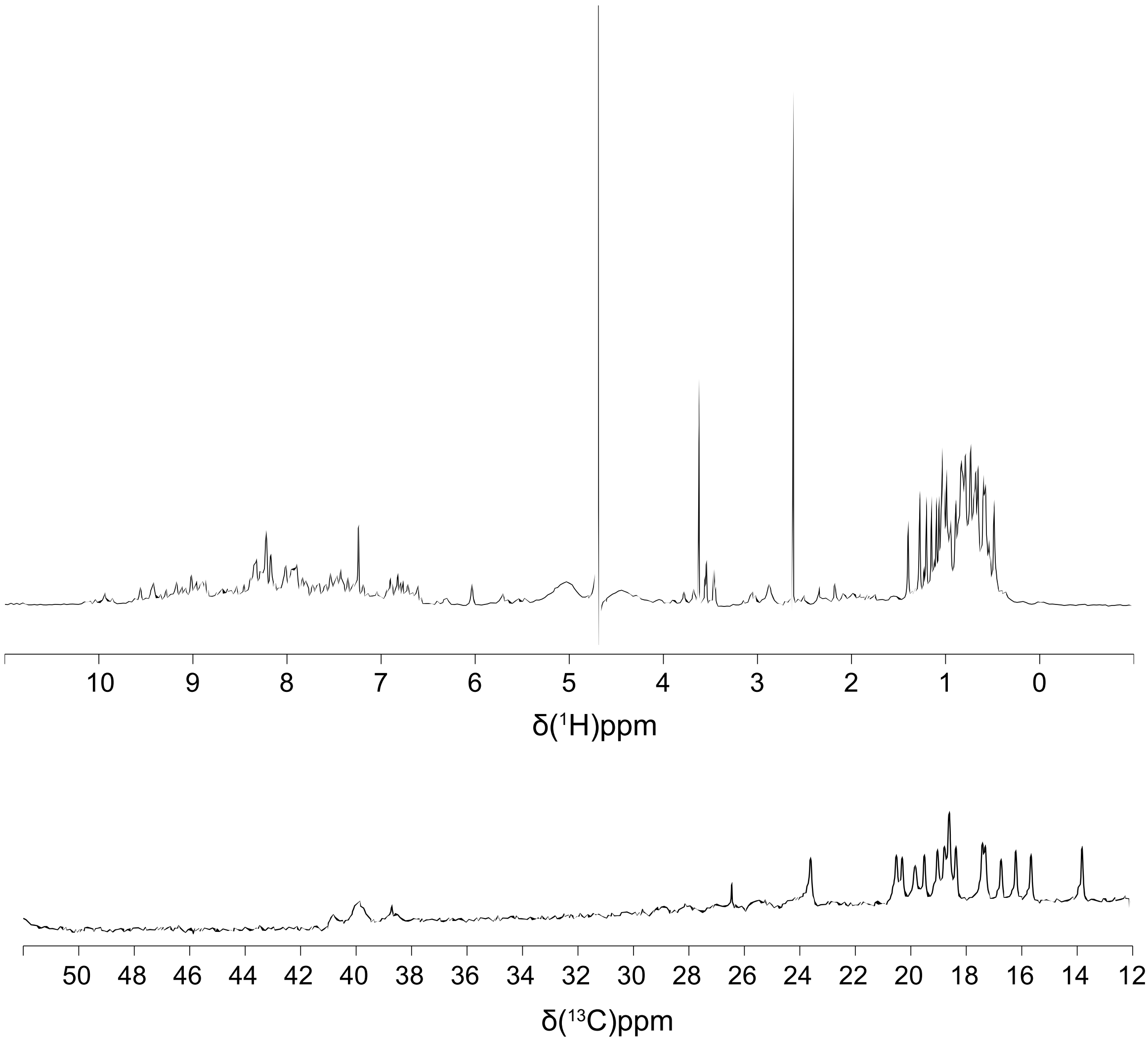

Figure S3. 1D ^{1}H- and ^{13}C-NMR spectra of PpiB expressed from 20 mL D_2O CFPS reaction using 800 mg eCells with 2-^{13}C-methyl-4-$^{2}H_3$-acetolactate. The sample is the same as that used for Figure 5B of the main text. Upper panel: ^{1}H-NMR spectrum recorded without ^{13}C-decoupling. The undeuterated methyl groups of valine between 0.5 and 1.5 ppm are the most prominent signals of the protein. Amide protons are observed due to some back-exchange during protein purification and measurement in 90 % H_2O/10 % D_2O. Lower panel: ^{13}C-NMR spectrum recorded with ^{1}H-decoupling. The spectra illustrate the high level of deuteration achieved.

Table S1. List of ^{2}H amino acids and their contribution to the cost of stereospecific perdeuterated eCell CFPS reaction to produce perdeuterated PpiB from 800 mg eCells in 20 mL CFPS buffer.[1]

Amino acid	CIL catalogue #	Price/g (USD)	Amount used (1 mM in 20 mL)	Cost of amount used (USD)
A	DLM-251-1	634	1.8 mg	1.1
R	DLM-541-0.1	11520	3.5 mg	40.3
G	DLM-280-5	50	1.5 mg	0.1
H	DNLM-7366-0.25	10396	3.1 mg	32.2
K	DLM-570-0.1	4160	2.9 mg	12.1
P	DLM-487-0.25	5200	2.3 mg	12.0
S	DLM-582-PK	1632	2.1 mg	3.4
T	DNLM-7367-0.5	4310	2.4 mg	10.3
V	DLM-488-PK	1244	N/A	N/A
N	DLM-6844-0.1	6770	2.6 mg	17.6
D	DLM-546-0.25	2056	2.7 mg	5.6
C	DLM-6901-0.1	10550	2.4 mg	25.3
E	DLM-556-0.1	4270	2.9 mg	12.4
Q	DNLM-6997-PK	5200	2.9 mg	15.1
L	DLM-567-PK	2120	2.6 mg	5.5
M	DLM-6797-PK	3530	3 mg	10.6
W	DLM-6903-0.25	6356	4.1 mg	26.1
Y	DNLM-7373-PK	4404	3.6 mg	15.9
I	DLM-141-0.25	4864	2.6 mg	12.6
F	DLM-372-PK	600	3.3 mg	2.0

[1] Prices from Cambridge Isotope Laboratories accessed 3rd April 2023. The total cost of the final column is USD 260.

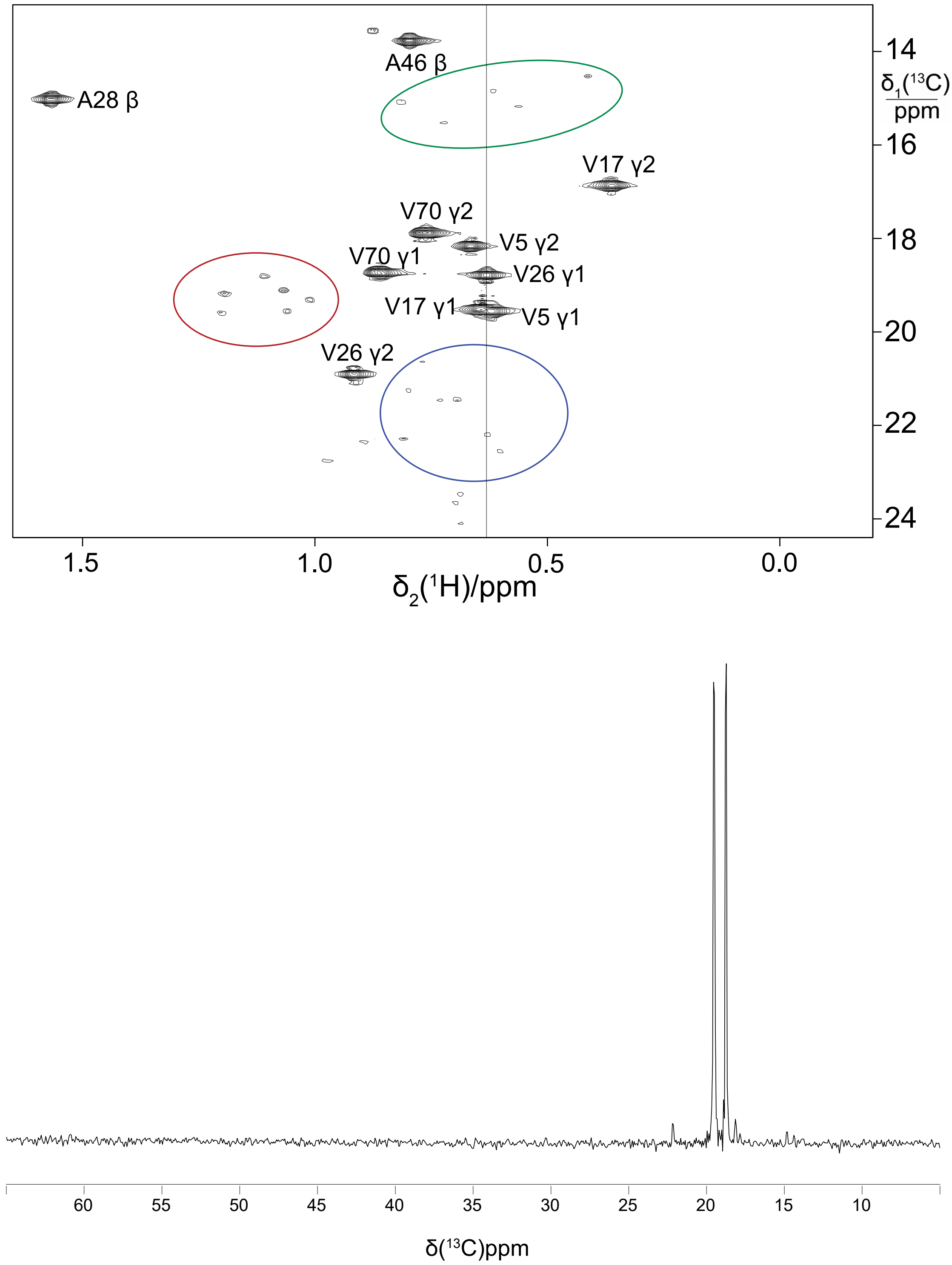

Figure S4. ^{13}C-HSQC spectrum of ubiquitin (Figure 3A in the main text) plotted at lower contour levels. The green, red and blue circles identify cross-peaks from isoleucine $C^{\delta1}H_3$, threonine $C^{\gamma}H_3$ and leucine $C^{\delta1/\delta2}H_3$ groups, respectively. Contours were plotted at levels increasing 1.4-fold between subsequent contour levels. Undesired cross-peaks are about 30-fold weaker than the expected cross-peaks. A vertical line marks the location, where the cross-section displayed in the lower panel was taken.

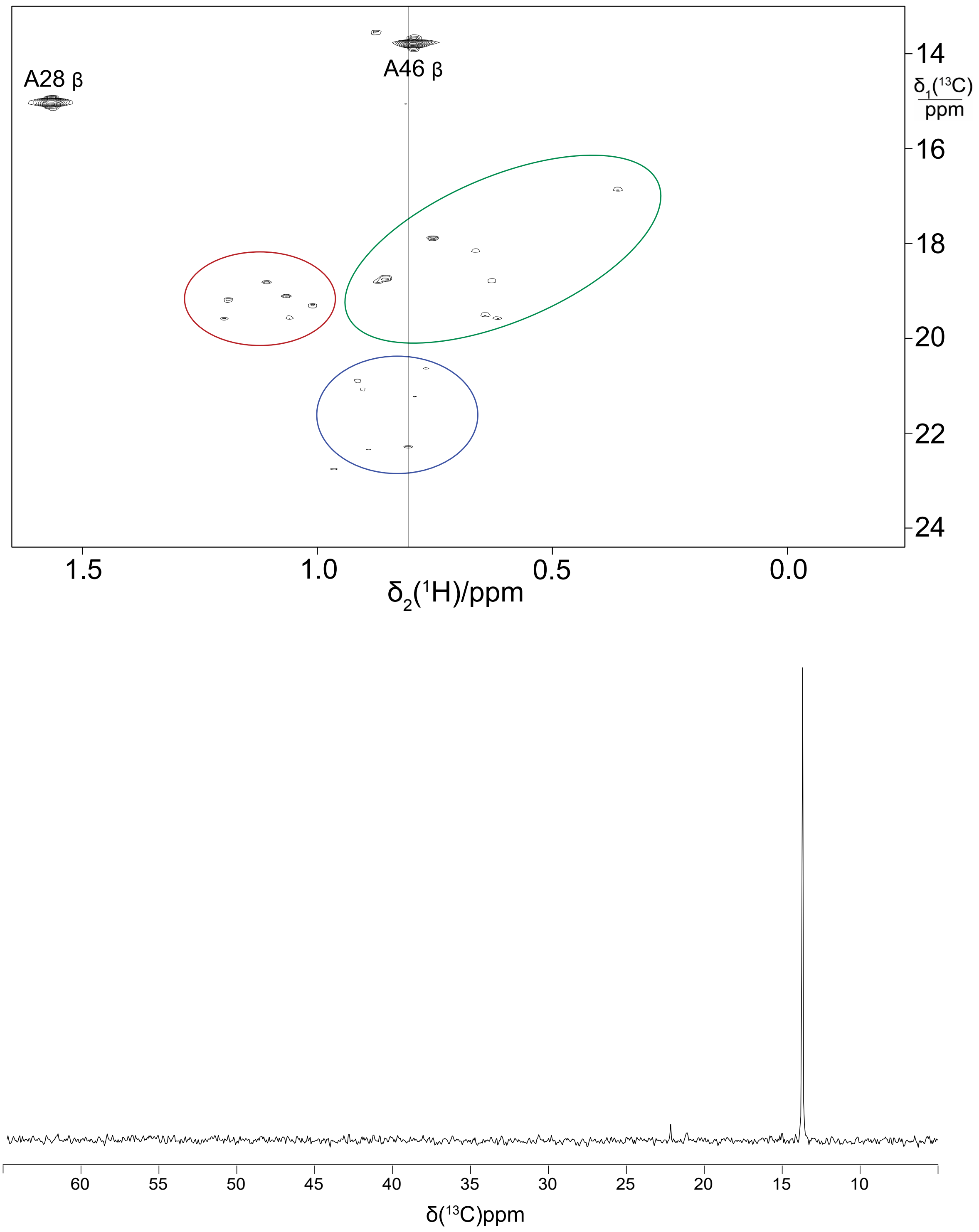

Figure S5. ^{13}C-HSQC spectrum of ubiquitin (Figure 3B in the main text) plotted at lower contour levels. The green, red and blue circles identify cross-peaks from valine $C^{\gamma1/\gamma2}H_3$, threonine $C^{\gamma}H_3$ and leucine $C^{\delta1/\delta2}H_3$ groups, respectively. The contour levels were plotted with a scaling factor of 1.4. Undesired cross-peaks are about 34-fold weaker than the expected cross-peaks. A vertical line marks the location of the cross section displayed in the lower panel.

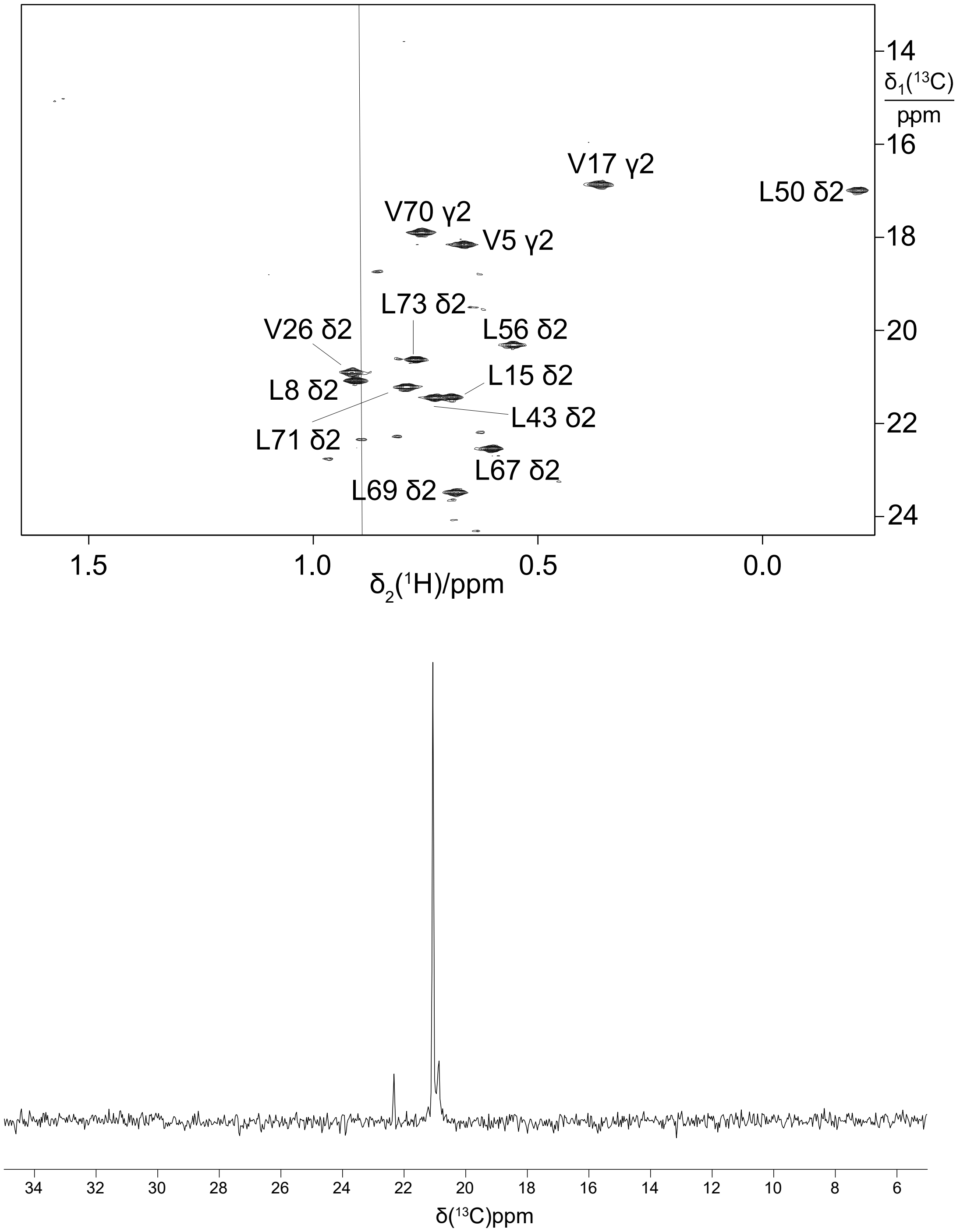

Figure S6. ^{13}C-HSQC spectrum of ubiquitin (Figure 4 in the main text) plotted at lower contour levels. Undesired cross-peaks are about 10-fold weaker than the expected cross-peaks. Subsequent contour levels were plotted with a scaling factor of 1.4. A vertical line marks the location of the cross-section shown in the lower panel.
